# Course of Fatigue and Sleep After Moderate to Severe Traumatic Brain Injury

**DOI:** 10.1097/HTR.0000000000001078

**Published:** 2025-07-22

**Authors:** Jessica Bruijel, Sven Stapert, Annemiek Vermeeren, Julie Staals, Jennie Ponsford, Caroline van Heugten

**Affiliations:** **Author Affiliations:** Department of Neuropsychology & Psychopharmacology, Faculty of Psychology and Neuroscience, Maastricht University, Maastricht, The Netherlands (Drs Bruijel, Stapert, Vermeeren, and Heugten); Limburg Brain Injury Centre, Limburg, The Netherlands (Drs Bruijel and Heugten); Department of Rehabilitation Adelante, Maastricht University Medical Centre+, Maastricht, The Netherlands (Dr Stapert); Department of Neurology, Maastricht University Medical Centre+, Maastricht, The Netherlands (Dr Staals); Turner Institute for Brain and Mental Health, School of Psychological Sciences, Monash University, Clayton, Victoria, Australia (Dr Ponsford); and Monash Epworth Rehabilitation Research Centre, Epworth Healthcare, Melbourne, Australia (Dr Ponsford).

**Keywords:** biopsychosocial model, fatigue, longitudinal study, sleep, traumatic brain injury

## Abstract

**Objective:**

To examine the development of different dimensions of fatigue and subjective and objective measures of sleep in the first 18 months post moderate to severe traumatic brain injury (TBI), and explore the association with biological (processing speed), psychological (mood), and social (restrictions in participation) factors across time.

**Participants:**

Forty-two participants with moderate-severe TBI (45 ± 16 years old, 33% female).

**Design:**

Longitudinal multicenter observational cohort study with 4 measurements (3, 6, 12, and 18 months post-injury).

**Main Measures:**

Dimensions of fatigue (Fatigue Severity Scale, FSS; Dutch Multifactor Fatigue Scale, DMFS), subjective (Pittsburgh Sleep Quality Index, PSQI) and objective sleep (actigraphy), processing speed (Symbol Digit Modalities Task, SDMT), mood (Hospital Anxiety and Depression Scale, HADS), and restrictions in participation (Utrecht Scale for Evaluation and Rehabilitation-Participation, USER-P).

**Results:**

Results showed reduced sleep quality (PSQI: poor sleep quality at 3 months 41%; 6 months 43%; 12 months 56%; 18 months 43%) and high levels of fatigue (FSS: severe fatigue at 3 months 41%; 6 months 38%; 12 months 33%; 18 months 34%) with no significant changes over time. Physical fatigue (DMFS: β = −0.11, *P* = .007) and total sleep time (β = −0.14, *P* = .015) decreased over time. More mood problems were associated with worse sleep quality (PSQI; β = 0.35, *P* = .021), shorter total sleep time (β = 0.14, *P* = .046), and higher levels of fatigue (FSS: β = 0.20, *P* = .036; DMFS-mental: β = 0.36, *P* = .028; DMFS-physical: β = 0.36, *P* = .029). Restrictions in participation were associated with fatigue but not with sleep.

**Conclusions:**

High and stable levels of fatigue and poor sleep quality in the first 18 months following moderate-severe TBI were found. These symptoms were associated with mood problems. Assessment and treatment of fatigue and sleep problems should be included in clinical practice. In line with other studies, we suggest that mood interventions might aid the treatment for fatigue and sleep quality.

TRAUMATIC BRAIN INJURY (TBI) is one of the most severe and debilitating neurological conditions, affecting on average of 326 per 100 000 people every year in Europe.^1^,^2^ While mild TBI often shows good recovery, moderate to severe TBI is more commonly linked with enduring consequences for both patients and their environment.^3^ Sleep problems and fatigue are key issues in the recovery process and negatively impact quality of life.^4^-^6^ Over half of the people with TBI have sleep problems^7^ and between 30% and 70% experience fatigue.^8^ However, findings regarding presence of fatigue and types of sleep problems are inconsistent, likely due to variations in study methodologies. Different studies include people at various time intervals post-injury, employ diverse injury severity parameters and use different measurement tools.^9^ Additionally, most studies use cross-sectional rather than longitudinal designs. This variability makes it challenging to compare results across studies and draw conclusions regarding the course of sleep problems and fatigue following TBI.^10^,^11^ Identifying the progression of sleep and fatigue over time may help pinpoint critical periods for implementing targeted interventions.

Longitudinal studies examining fatigue and sleep in TBI populations are rare and have primarily focused on mild TBI^12^,^13^ or mixed mild and moderate-severe TBI populations.^14^-^16^ These studies have mostly relied on subjective measures, despite evidence of discrepancies between subjective and objective assessments of sleep in people with TBI.^17^ Furthermore, various dimensions of fatigue (eg, physical and mental fatigue) are often overlooked, despite the likelihood that they follow distinct trajectories and may require different treatment strategies. Consequently, it is important to assess different dimensions of fatigue and include both subjective and objective sleep measures.^18^ Outcomes and prognosis following TBI vary significantly among individuals, regardless of injury severity.^9^ This variability implies that outcome is not only influenced by biological factors, but should be studied in a biopsychosocial model in which physical, cognitive, affective and social factors interact with sleep and fatigue.^6^,^8^,^9^

This study addresses these limitations of earlier studies by examining sleep and fatigue concurrently in a longitudinal design incorporating different dimensions of fatigue and both subjective and objective measures of sleep to better understand their development following specifically moderate-severe TBI. Biopsychosocial factors were explored over time to assess their relationship with post-TBI fatigue and sleep problems. Understanding these complex interactions is crucial for managing fatigue and sleep problems associated with TBI. From a strict biological perspective, it can be hypothesized that sleep problems and fatigue decrease over time due to natural recovery following TBI. However, other factors may influence these symptoms; for instance, worrying about recovery could increase sleep problems over time. It was hypothesized that the associations between biopsychosocial factors and post-TBI fatigue and sleep problems would change over time. Specifically, it was expected that associations with biological factors would be strongest in the first 6 months in line with TBI recovery and then decline, while associations with psychological and social factors would gradually increase and become more apparent between 12 and 18 months.

## METHODS

### Participants

The protocol for this study has been previously published. All participants provided written informed consent. The Medical Ethics Committee of University Hospital Maastricht/Maastricht University (NL60322.068.17) and all participating centers approved the study protocol. People with moderate to severe TBI were recruited in several hospitals and rehabilitation clinics across the Netherlands between October 2017 and December 2022. Participants were attended to the study by their treating physician, who conducted an initial eligibility check. Participants with a clinically confirmed diagnosis of a first moderate to severe, closed head TBI defined as Glasgow Coma Scale (GCS) score <13;^19^ or post-traumatic amnesia (PTA) >24 hours; or trauma related intracranial neuroimaging abnormalities; or loss of consciousness (LOC) >30 min were included.^20^ Additional inclusion criteria were age between 21 and 80 years and fluency in Dutch. Exclusion criteria were a prior moderate-severe TBI diagnosed by a neurologist or a concussion in last half year, presence of another neurological condition, sleep-wake disturbance or fatigue due to another medical condition than TBI, history of alcohol and/or drug abuse, prior mental disorder (requiring treatment), pregnancy, and inability to complete questionnaires based on clinical judgment.

### Procedure

The study started with a screening visit, where eligibility was assessed through a structured interview, preferably within the first 6 weeks after injury in which consent was signed and demographics and pre-injury characteristics (age, sex, education, marital status, level of occupational achievement, psychological and medical history, pre-TBI sleep problems (PSQI)) were collected. Information regarding the injury was retrieved from the hospital database. The follow-up visits took place at approximately 3 months (V1), 6 months (V2), 12 months (V3) and 18 months (V4) post injury, within 2 weeks before or after follow-up date. Visits took place at Maastricht University, a participating clinical institutes or the participant’s home. Visits were preferably scheduled between 11:00 and 15:00 h to minimize effects of the circadian rhythm. In the week before the visits, the participant wore an actigraph and completed a sleep diary for 7 days at home (daily living). The study is registered at CCMO (trial 50267).

### Measures

All measures have good psychometric properties and have been used in the TBI population before, see protocol publication for details.^21^

### Outcome measures

#### Dimensions of fatigue

Subjective fatigue was measured with 2 questionnaires, the Fatigue Severity Scale (FSS), and the Dutch Multi-Factor Fatigue Scale (DMFS) which assesses multidimensional aspects of fatigue. The FSS measures the impact of fatigue on activities of daily life and distress caused by fatigue.^22^ Subsequent psychometric research on the FSS showed improved validity and sensitivity to changes in stroke populations when the first 2 items were removed.^23^ Therefore, the current study used the average score of items 3-9, rated on a 7-point Likert scale. The DMFS is a newly developed questionnaire that examines several factors of fatigue following TBI, including impact of fatigue, mental fatigue, signs and direct consequences of fatigue, physical fatigue and coping with fatigue.^24^ For both scales a higher score indicates more severe fatigue and for FSS a mean score ≥4 indicates severe fatigue.^22^

#### Subjective sleep quality

Subjective sleep quality was assessed with the Pittsburg Sleep Quality Index (PSQI). The PSQI examines 7 components namely, overall sleep quality, sleep onset latency (SOL), total sleep time (TST), sleep efficiency, sleep disturbances, use of sleep medication and daytime dysfunction. The global score is calculated by adding the 7 component scores. The questionnaire can discriminate between “good” and “poor” sleepers, with a global score of >5 indicating poor sleep quality.

#### Objective sleep

Sleep problems were examined objectively using an actigraph, which measured sleep-wake patterns during 1 week (GENEActiv, Activinsights Ltd., Cambridgeshire, UK). The actigraph is a wrist-watch-like device, worn continuously on the non-dominant wrist, which allows the participant to continue normal routines in the natural environment. Actigraphy has shown to be a satisfactory objective estimate of sleep especially for global sleep parameters including TST and wake after sleep onset (WASO).^25^ Questions about bed and wake times,^26^ were used for preprocessing of actigraphy data (ie, time window for algorithm to determine sleep/wake and SOL and sleep efficiency). The diary was completed in the morning and was filled out for 7 consecutive days concurrent with the actigraphy.

### Predictors

#### Processing speed

Symbol Digit Modalities Test (SDMT) measures attention and processing speed. The SDMT is sensitive to impairments of speed of information processing following TBI.^27^ The extent to which cognitive functioning is affected was used in this study as a proxy for the severity of brain injury.^28^ Norm scores adjusted for age and education were used to predictor fatigue and sleep.

#### Emotional distress

Level of emotional distress was examined with the Hospital Anxiety and Depression Scale (HADS). The HADS includes 2 subscales measuring anxiety and depression. A subscale score of ≥8 is an indicator of symptoms of depression or anxiety also in people with TBI.^29^ Higher score denote more psychological distress. This study used the total score of the HADS, presenting overall emotional distress, to predict fatigue and sleep.

#### Restrictions in participation

The Utrecht Scale for Evaluation and Rehabilitation-Participation (USER-P)^30^ was used to assess restrictions in participation. The questionnaire measures 3 aspects of participation: frequency of behaviors, experienced participation restrictions due to health condition, and satisfaction with participation. Higher scores indicate good levels of participation (higher frequency, less restrictions, higher satisfaction). This study only included the participation restrictions scale to predict fatigue and sleep.

### Statistical analyses

Statistical analyses were performed in R (version 4.3.1^31^; and figures were made using the ggplot2 package).^32^ To examine changes over time in different dimensions of fatigue and subjective and objective measures of sleep, linear mixed-effect regression models using the lme4 package were performed. These models included time as predictor, with subject as random intercept to cluster observations within a subject. In case time was a significant predictor, simple contrasts comparing consecutive time points were used to determine how fatigue or sleep changed over time using estimated marginal means with Tukey’s honestly significant difference test to adjust for the risk of false discoveries consequent to multiple comparisons (emmeans package). Alternative forms of change over time were examined by modelling Time as polynomial terms (linear, quadratic, cubic) and comparing the models using Likelihood Ratio Tests and Akaike Information Criterion. Next, similar analyses explored changes over time in the factors of the biopsychosocial model. Exploratory analyses were performed to examine associations between biopsychosocial factors (processing speed, mood and restrictions in participation) and outcome variables (dimensions of fatigue and subjective and objective sleep measures) across time using linear mixed-effect regression models. It was first determined whether these associations changed across the 4 time points (ie, time by predictor interactions). In case of a significant interaction, simple interaction contrasts comparing consecutive time points were used to determine whether the association decreases or increases. Predictors were centered to reduce multicollinearity. Due to COVID-19 restrictions, SDMT data were missing from the period when face-to-face appointments could not be conducted. To maximize the dataset and maintain statistical power, missing values were imputed using predictive mean matching (mice package in R).

## RESULTS

In total 47 participants were included in the study. Five participants were excluded or dropped out after the screening visit, leaving data of 42 participants for the analyses. The screening visit took place on average 7 weeks after TBI (SD: 4.7 weeks, range 2.6-25.6 weeks). Demographic characteristics at time of inclusion and injury characteristics are presented in Table 1. Participants were on average 45 years old at inclusion and 33% were female. All participants showed trauma related intracranial neuroimaging abnormalities on CT/MRI scan and were therefore classified as moderate-severe TBI.^20^ Over half the participants sustained TBI due to a bike-related accident. Participants were all living independently at 3 months post-injury. Poor sleep quality prior to TBI was reported by 31% of participants. In total 31 participants completed all measures (see Table 2 for number of participants per visit).

**TABLE 1.** Demographic characteristics at the time of inclusion and injury characteristics (N = 42)

	Mean ± SD/Percentage	Min-Max/N
Age	45.2 ± 16.7	19-70
Sex (female)	33.3%	14
Marital status		
Married/cohabitating	50.0%	21
Without partner	50.0%	21
Living situation at inclusion		
Independent	92.9%	39
Residential facility	7.2%	3
Employment (before TBI)		
Employed	81.0%	34
Unemployed/ retired	19.0%	8
Hours of paid work per week if employed (before TBI)	34.4 ± 9.2	3-40
Finished higher education^a^	30.9%	13
Psychological treatment past (yes)	33.3%	14
Cause injury		
Fall	26.2%	11
Motor vehicle	16.7%	7
Violence	2.4%	1
Fall off bike/ bike vs car	52.4%	22
Sports	2.4%	1
Injury characteristics^b^		
Duration loss of consciousness (hours) (N = 18)	25.8 ± 78.7	0-336
Duration post-traumatic amnesia (days) (N = 13)	4.2 ± 4.4	0-12
Glasgow Coma Scale (N = 28)	11.0 ± 4.3	3-15
Abnormalities brain CT/MRI scan (N = 39)	100%	39
Epidural Hematoma (N = 38)	18.4%	7
Subdural Hematoma (N = 39)	43.6%	17
Subarachnoid Hemorrhage (N = 39)	53.8%	21
Subgaleal Hematoma (N = 38)	2.6%	1
Intracerebral Hemorrhage (N = 38)	13.1%	5
Contusion (N = 39)	71.8%	28
Other injuries		
Physical Injury (N = 38)	28.9%	11
Organ injury (N = 38)	7.9%	3

^a^ Higher education level includes completion of a college or university degree.

^b^ Information was often missing from the medical files therefore total N is reported for each measure.

**TABLE 2.** Results of the dimensions of fatigue, subjective and objective sleep measures and predictors of the biopsychosocial model at the 4 time points

	Visit 1	Visit 2	Visit 3	Visit 4	Stats
	3 months	6 months	12 months	18 months	
	Mean ± SD [min-max]	Mean ± SD [min-max]	Mean ± SD [min-max]	Mean ± SD [min-max]	Standardized beta and confidence interval
	N = 36	N = 38	N = 37	N = 37	
Fatigue					
FSS—7	3.4 ± 1.6 [1.1-6.1]	3.3 ± 1.5 [1.0-6.3]	3.3 ± 1.6 [1.0-6.9]	3.2 ± 1.6 [1.0-6.4]	β = −0.06 [CI −0.15 to 0.02], *P* = .13
Clinically significant ≥4	16 (44.4%)	15 (39.5%)	10 (27.8%)	13 (35.1%)	
DMFS—total	104.5 ± 22.2 [65.0-148.0]	104.3 ± 25.7 [46.0-150.0]	101.5 ± 24.0 [51.0-150.0]	101.7 ± 25.2 [51.0-145.0]	β = −0.05 [CI −0.13 to 0.03], *P* = .23
DMFS—impact of fatigue	28.4 ± 9.6 [14.0-50.0]	29.0 ± 10.1 [11.0-47.0]	27.4 ± 9.3 [13.0-48.0]	27.5 ± 10.0 [12.0-44.0]	β = −0.06 [CI −0.15 to 0.03], *P* = .17
DMFS—mental fatigue	22.8 ± 4.5 [13.0-31.0]	21.6 ± 5.7 [8.0-31.0]	21.2 ± 6.1 [8.0-32.0]	21.5 ± 5.2 [8.0-32.0]	β = −0.09 [CI −0.19 to −0.00], *P = .053*
DMFS—signs of fatigue	23.2 ± 6.4 [12.0-35.0]	23.8 ± 7.4 [9.0-39.0]	23.9 ± 6.4 [9.0-38.0]	23.9 ± 6.6 [9.0-35.0]	β = 0.05 [CI −0.04 to 0.15], *P* = .27
DMFS—physical fatigue	15.0 ± 3.7 [9.0-21.0]	15.6 ± 4.2 [8.0-23.0]	14.3 ± 3.9 [8.0-25.0]	14.1 ± 4.6 [7.0-23.0]	**β = −0.11 [CI −0.20** to **−0.03], *P* = .007**
DMFS—coping with fatigue	15.1 ± 3.8 [7.0-24.0]	14.3 ± 3.3 [8.0-22.0]	14.6 ± 3.1 [9.0-23.0]	14.6 ± 3.9 [8.0-23.0]	β = −0.01 [CI −0.13 to 0.11], *P* = .89
Sleep					
PSQI	5.2 ± 2.3 [1.0-10.0]	5.4 ± 3.0 [0.0-16.0]	5.9 ± 3.2 [1.0-15.0]	5.4 ± 2.9 [1.0-13.0]	β = −0.02 [CI −0.12 to 0.07], *P* = .60
Clinically significant >5	14 (38.9%)	17 (44.7%)	20 (55.5%)	16 (43.3%)	
Actigraphy/ sleep diary	N = 33	N = 32	N = 33	N = 36	
Bedtime (time of Day)	24:03 ± 0.59 [22:32-02:16]	24.08 ± 1.10 [20:46-02:22]	24:12 ± 1.02 [22:41-02:37]	24:09 ± 1.07 [22:13-03:50]	β = 0.02 [CI −0.08 to 0.11], *P* = .72
Wake time (time of Day)	7:32 ± 1.08 [5:15-9:41]	7:34 ± 1:16 [5:35-10:10]	7:21 ± 1.13 [5:19-10:12]	7:16 ± 1.19 [5:10-11:45]	β = −0.08 [CI −0.18-0.02], *P* = .10
Total sleep time (hours)	6.9 ± 0.8 [5.7-8.4]	6.8 ± 0.8 [5.1-8.1]	6.6 ± 1 [4.2-8.1]	6.5 ± 0.9 [4.4-8.5]	**β = −0.14 [CI −0.26** to **−0.03], *P* = .015**
WASO (minutes)	34 ± 19.9 [8.9-87.4]	36.6 ± 21.8 [11.2-100.9]	31.6 ± 14.4 [10.7-68.2]	34.4 ± 23.0 [8.8-141.9]	β = 0.00 [CI −0.13 to 0.13], *P* = .98
SOL (minutes)	17.7 ± 24.2 [2.3-110]	15.9 ± 14.7 [1.9-62.3]	18.7 ± 21.6 [0.4-79.6]	14.8 ± 15.5 [1.3-81.8]	β = −0.05 [CI −0.20 to 0.11], *P* = .55
Sleep efficiency (%)	87.6 ± 7.4 [66.9-96.2]	86.7 ± 7 [68.5-96.3]	86.2 ± 7.4 [60.5-96]	86.5 ± 7.1 [62.4-96]	β = −0.05 [CI −0.18 to 0.08], *P* = .45
TIB (hours)	8.9 ± 0.8 [7.3-10.8]	9.0 ± 1.3 [6.9-13.4]	8.7 ± 1 [7.2-11.2]	8.7 ± 1.2 [7.0-13.0]	β = −0.09 [CI −0.22 to 0.04], *P* = .17
Number of days	6.6 ± 0.8 [3-7]	6.8 ± 0.6 [5-7]	6.6 ± 0.7 [5-7]	6.5 ± 0.8 [4-7]	β = −0.08 [CI −0.26 to 0.09], *P* = .34
Predictors					
*SDMT*	N = 34 51.5 ± 11.5 [20.0-70.0]	N = 35 53.1 ± 12.4 [25.0-75.0]	N = 30 58.7 ± 13.5 [30.0-80.0]	N = 35 55.9 ± 11.7 [35.0-80.0]	**β = 0.16 [CI 0.08** to **0.24], *P* < .001**
*HADS—total*	10.0 ± 6.4 [0.0-28.0]	10.0 ± 6.6 [0.0-26.0]	10.2 ± 6.2 [0.0-27.0]	10.4 ± 6.7 [0.0-25.0]	β = 0.03 [CI −0.05 to 0.10], *P* = .51
HADS—depression	4.4 ± 3.7 [0.0-13.0]	4.2 ± 3.5 [0.0-12.0]	4.7 ± 3.7 [0.0-16.0]	4.9 ± 3.9 [0.0-16.0]	β = 0.05 [CI −0.03 to 0.14], *P* = .21
Clinically significant ≥8	6 (16.7%)	8 (21.1%)	7 (19.4%)	7 (18.9%)	
HADS—anxiety	5.6 ± 3.7 [0.0-15.0]	5.8 ± 3.8 [0.0-16.0]	5.6 ± 3.2 [0.0-11.0]	5.5 ± 3.6 [0.0-14.0]	β = −0.01 [CI −0.09 to 0.07], *P* = .85
Clinically significant ≥8	9 (25.0%)	13 (34.2%)	10 (27.8%)	12 (32.4%)	
*USER-P—restrictions*	84.7 ± 14.5 [52.4-100.0]	87.9 ± 13.4 [53.3-100.0]	92.0 ± 12.2 [53.3-100.0]	91.5 ± 12.3 [44.4-100.0]	**β = 0.22 [CI 0.15** to **0.30], *P* < .001**
USER-P—frequency	35.4 ± 6.8 [11.4-46.4]	39.1 ± 9.3 [15.0-53.9]	40.3 ± 6.9 [19.6-52.1]	37.8 ± 9.8 [6.8-54.3]	β = 0.09 [CI −0.04 to 0.22], *P* = .17
USER-P—satisfaction	71.9 ± 16.1 [35.0-100.0]	72.1 ± 14.7 [41.7-96.9]	72.9 ± 14.6 [38.9-95.0]	73.9 ± 15.1 [30.0-97.5]	β = 0.05 [CI −0.04 to 0.15], *P* = .28

If data were missing, the number of participants that data were available for is reported. Significant changes over time are indicated in bold. m*smaller N because of no face-to-face appointments due to COVID; CI, confidence interval; FSS, Fatigue Severity Scale, 7-items; DMFS, Dutch Multi-Factor Fatigue Scale; PSQI, Pittsburgh Sleep Quality Index; WASO, wake after sleep onset; SOL, sleep onset latency; TIB, time in bed; HADS, Hospital Anxiety and Depression Scale; USER-P, Utrecht Scale for Evaluation and Rehabilitation-Participation.

### Change in symptoms over time

**Figure 1.**
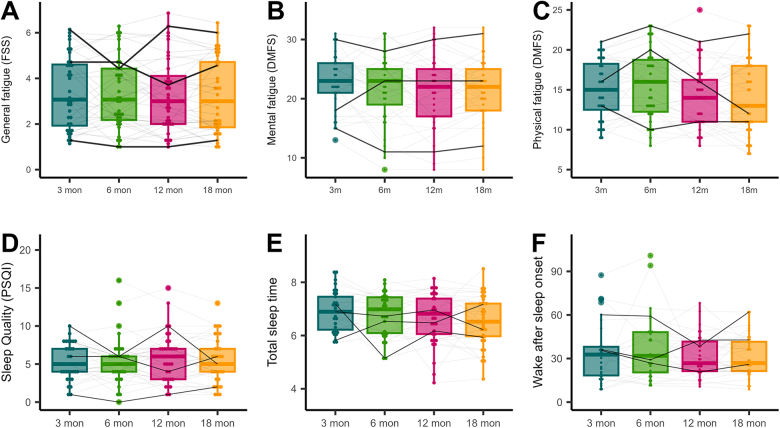
Trajectories of the dimensions of subjective fatigue and subjective and objective sleep measures in the first 18 months post traumatic brain injury. Three participants are made bold in each graph to show fluctuations in symptoms over time. Abbreviations: FSS, Fatigue Severity Scale; DMFS, Dutch Multifactorial Fatigue Scale; PSQI, Pittsburgh Sleep Quality Index.

Results for changes in the first 18 months post-TBI in dimensions of fatigue, subjective and objective sleep (Figure 1), processing speed, mood, and participation restriction are presented in Table 2. Physical fatigue (DMFS, β = −0.11, *P* = .007) and TST (actigraphy, β = −0.14, *P* = .015) decreased over time. Post hoc analyses showed that physical fatigue significantly decreased between 6 and 12 (estimate = 1.22, *P* = .049), and 6 and 18 months post-injury (estimate = 1.39, *P* = .017). For TST there was a trend toward a significant decrease between 3 and 18 months post-injury (estimate = 0.34, *P* = .088). There was a trend toward a decrease in mental fatigue over time (DMFS, β = −0.09, *P* = .053). General fatigue (FSS), subjective sleep quality (PSQI) and other subjective/objective sleep parameters did not significantly change over time. However, as illustrated in Figure 1, the trajectories of fatigue and sleep over time varied among participants. Levels of fatigue (FSS≥4) were moderately high at all 4 time points with 44% experiencing severe fatigue at 3 months, 40% at 6 months, 28% at 12 months, and 35% at 18 months. Poor sleep quality was present in around half of participants at all 4 time points, with 39% experiencing poor sleep quality at 3 months, 45% at 6 months, 56% at 12 months and 43% at 18 months. Around 17%, 18%, 22%, and 14% had sleep problems before the TBI and at 3, 6, 12, and 18 months, respectively. Both fatigue and sleep problems were present in 22%, 26%, 19%, and 24% of participants at 3, 6, 12, and 18 months post-injury, respectively. Recommended sleep efficiency above 85% on average was present in 82% at 3 months but decreased over time to 75%, 70%, and 67% at 6, 12, and 18 months, respectively. There were only linear changes in sleep and fatigue over time (Supplementary Digital Content, Tables S1 and S2, available at: http://links.lww.com/JHTR/A960). Results were consistent when including only participants who completed all 4 measures (Supplementary Digital Content, Table S3, available at: http://links.lww.com/JHTR/A960).

Restrictions in participation (USER-P, β = 0.22, *P* < .001) showed a linear decrease over time and additional quadratic changes over time (β = −1.00, *P* = .027). Post-hoc analyses indicated a significant decrease between 3 and 12 (estimate = −8.27, *P* < .001), 3 and 18 (estimate = −7.51, *P* < .001), 6 and 12 (estimate = −4.54, *P* = .006), and 6 and 18 months post-injury (estimate = −3.83, *P* = .028). Processing speed improved over time (SDMT, β = 0.16, *P* < .001) and showed an additional cubic change over time (β = −1.27, *P* = .004). There was a change between 3 and 12 (estimate = −6.46, *P* < .001), and 6 and 12 months post-injury (estimate = −5.51, *P* < .001). Mood problems showed no change over time. Frequency in participation showed only a quadratic change over time (β = −2.17, *P* = .004). Around one-fifth of participants experienced at least mild symptoms of depression at each time point (mild: 3%-18%, moderate: 3%-14%, severe 0%-3%). Participants experienced more symptoms of anxiety, with around a quarter of participants experiencing at least mild symptoms of anxiety at 3 and 12 months post-injury and around one-third at 6 and 18 months post-injury (mild: 11%-24%, moderate: 8%-14%, severe 0%-3%).

### Association between biopsychosocial factors and fatigue and sleep

Exploratory analyses examining the association between processing speed (bio), mood (psycho), participation restrictions (social), and dimensions of subjective fatigue are presented in Table 3 (Figure S1, available at: http://links.lww.com/JHTR/A960). More mood problems were associated with all dimensions of fatigue, general (β = 0.20, *P* = .036), physical (β = 0.36, *P* = .029) and mental fatigue (β = 0.36, *P* = .028) and these associations did not change over time. More restrictions in participation were associated with general (β = −0.42, *P* = .013) and physical fatigue (β = −0.34, *P* = .039) and these associations showed no change over time. The association between participation restrictions and mental fatigue showed a trend toward changing over time (β = −0.10, *P =* .095), this association seemed stronger in 6 to 18 months post-injury compared to 3 months (Supplementary Digital Content, Figure S1, available at: http://links.lww.com/JHTR/A960). There was a trend toward a change in the association between processing speed and physical fatigue over time (β = 0.08, *P* = .073), this associated seemed stronger in the first 6 months following injury (Supplementary Digital Content, Figure S1, available at: http://links.lww.com/JHTR/A960). Processing speed was not associated with general or mental fatigue (all *p*’s >.20)

**TABLE 3.** The association between the factors of the biopsychosocial model and dimensions of fatigue

	General fatigue (FSS)			Physical fatigue (DMFS)			Mental fatigue (DMFS)		
Marginal R^2^/Conditional R^2^	0.34/0.72			0.40/0.77			0.30/0.69		
*Predictors*	*Std. beta*	*Standardized CI*	*P*	*Std. beta*	*Standardized CI*	*P*	*Std. beta*	*Standardized CI*	*P*
Time	0.03	−0.06 to 0.13	.46	−0.04	−0.13 to 0.04	.33	−0.06	−0.16 to 0.04	.26
Processing speed (SDMT)	−0.07	−0.23 to 0.08	.62	−0.01	−0.16 to 0.13	.13	0.04	−0.12 to 0.20	.88
Mood (HADS)	0.20	0.05 to 0.36	**.036**	0.36	0.21 to 0.51	**.029**	0.36	0.19 to 0.52	**.028**
Participation restrictions (USER-P)	−0.42	−0.58 to −0.25	**.013**	−0.34	−0.50 to −0.19	**.039**	−0.25	−0.43 to −0.08	.82
Time × processing speed	−0.06	−0.16 to 0.03	.020	0.08	−0.01 to 0.17	*.073*	0.01	−0.10 to 0.11	.89
Time × mood	−0.04	−0.14 to 0.06	.45	0.04	−0.05 to 0.13	.42	0.01	−0.09 to 0.12	.79
Time × participation restrictions	−0.06	−0.15 to 0.06	.44	−0.04	−0.14 to 0.06	.42	−0.10	−0.22 to 0.02	*.095*

FSS, Fatigue Severity Scale; SDMT, Symbol Digit Modality Task; HADS, Hospital Anxiety and Depression Scale; USER-P, Utrecht Scale for Evaluation and Rehabilitation-Participation.

The association between the biopsychosocial factors and subjective and objective sleep quality are presented in Table 4 and Supplementary Digital Content, Figure S2, available at: http://links.lww.com/JHTR/A960. More mood problems were associated with worse subjective sleep quality (PSQI; β = 0.35, *P* = .021) and this association did not change over time. The association between mood and TST changed over time (β = 0.14, *P* = .046, Supplementary Digital Content, Figure S2, available at: http://links.lww.com/JHTR/A960). This association seemed negative at 3 months but this was not significant (β = −14.2, *P* = .11) and was not present at the other time points (all *p*’s >.86). Further post-hoc analyses did not show a significant difference in this association between specific time points. WASO was not associated with mood. Processing speed and restrictions in participation were not associated with subjective or objective sleep measures. Sensitivity analyses excluding the missing SDMT data, showed similar results (Supplementary Digital Content, Tables S4 and S5, available at: http://links.lww.com/JHTR/A960). Adding age, sex and pre-TBI sleep problems as covariates to the models did not change the results.

**TABLE 4.** The association between the factors of the biopsychosocial model, and subjective and objective sleep

	Sleep quality (PSQI)			TST (actigraphy)			WASO (actigraphy)		
Marginal R^2^/Conditional R^2^	0.20/0.73			0.07/0.63			0.07/0.64		
*Predictors*	*Std. beta*	*Standardized CI*	*P*	*Std. beta*	*Standardized CI*	*P*	*Std. beta*	*Standardized CI*	*P*
Time	−0.02	−0.12 to 0.08	.73	−0.17	−0.30 to −0.04	**.012**	0.00	−0.12 to 0.13	.96
Processing speed (SDMT)	0.14	−0.03 to 0.32	.17	−0.12	−0.33 to 0.09	.35	−0.10	−0.31 to 0.10	.73
Mood (HADS)	0.35	0.17 to 0.52	**.021**	0.08	−0.14 to 0.29	.21	0.26	0.05 to 0.47	.095
Participation restrictions (USER-P)	−0.17	−0.36 to 0.02	.24	0.24	0.02 to 0.47	.87	0.01	−0.22 to 0.23	.68
Time × processing speed	−0.03	−0.14 to 0.07	.56	0.03	−0.11 to 0.16	.69	−0.02	−0.15 to 0.11	.80
Time × mood	−0.00	−0.11 to 0.11	.99	0.14	0.00 to 0.28	**.046**	−0.02	−0.16 to 0.11	.74
Time × participation restrictions	0.00	−0.12 to 0.12	.99	0.12	−0.03 to 0.27	.11	0.04	−0.11 to 0.18	.63

Based on 147/134 observations clustered within 42 participants for PSQI and actigraphy respectively. The marginal R2 represents the proportion of variance explained by fixed effects. The conditional R2 represents the proportion of variance explained by both fixed and random effects. CI, confidence interval; PSQI, Pittsburgh Sleep Quality Index; TST, total sleep time; WASO, wake after sleep onset; SDMT, Symbol Digit Modality Task; HADS, Hospital Anxiety and Depression Scale; USER-P, Utrecht Scale for Evaluation and Rehabilitation-Participation.

## DISCUSSION

This study examined the course of different dimensions of fatigue and subjective and objective measures of sleep at 4 time points in the first 18 months following moderate to severe TBI. Levels of general and mental of fatigue and poor subjective sleep quality were moderate to high and stable over this time-period. There was only a decrease in physical fatigue and objective TST over time. Other objective measures of sleep, such as sleep fragmentation (ie, WASO) and SOL did not show changes over time. The association between factors of the biopsychosocial model and, fatigue and sleep did not change over time. More mood problems (ie, symptoms of anxiety and depression) and more restrictions in participation were associated with higher levels of fatigue. More mood problems were also associated with poorer subjective sleep quality and a shorter sleep duration. Processing speed improved over time, but was not significantly associated with different dimensions of fatigue, subjective sleep quality, or sleep duration and fragmentation. In addition, participants experienced fewer restrictions in participation over time.

Across the entire participant group, no significant changes were observed over time in general fatigue, mental fatigue, subjective sleep quality, or sleep fragmentation. Moderate to severe fatigue affected 28-44% of participants, while poor sleep quality was present in 39-55%. Notably, nearly half of the participants who experienced sleep problems reported having similar issues prior to their TBI. The absence of significant changes in fatigue aligns with the limited longitudinal research available on TBI, which similarly reported minimal changes in fatigue over time.^16^,^33^,^34^ Longitudinal studies of sleep quality following TBI are scarce and report mixed results, with the majority focusing on mild TBI cases.^13^,^33^,^35^ Our results suggest that both fatigue and poor sleep quality remain persistent challenges for individuals with TBI in first 18 months post-injury. This finding underpins the necessity to treat fatigue and sleep problems after TBI as these symptoms will not resolve over time. Nevertheless, visual inspection of individual trajectories revealed considerable variability and symptom fluctuations, which is consistent with findings from studies in broader brain injury populations.^36^,^37^ Therefore, despite the absence of statistically significant group-level changes in most fatigue and sleep measures, symptom fluctuations at the individual level underscore the importance of more personalized treatment strategies. Furthermore, given that both fatigue and sleep problems negatively impact participation in daily activities,^14^ these results highlight the need for early identification, continuous monitoring of individuals at higher risk, and timely, tailored rehabilitation programs.

Physical fatigue and TST both showed a decline in the 18 months post-TBI. The reduction in physical fatigue may be explained by improvements in physical functioning during the first year following injury.^33^,^34^ The decrease in TST is in line with other studies that show an increase in sleep duration in the acute phase post-injury.^38^-^40^ The increased need for sleep following brain injury has been attributed to factors such as loss of wake-promoting histaminergic cells in the hypothalamic tuberomammillary nucleus^41^ and neuroplasticity-related recovery processes.^39^ Furthermore, our findings suggest a shift in the relationship between sleep duration and mood over time. While more mood problems seemed linked to a shorter sleep duration in the subacute phase (3 months post-injury), this association weakened in the chronic phase. The interplay between mood and sleep duration may grow more complex over time, as mood fluctuations can result in varying sleep patterns—both reduced and prolonged^42^—while other factors like lifestyle and chronic pain may further complicate this relationship.^34^ Although TST decreased over time, subjective sleep quality did not show a corresponding decline. This may be explained as poor subjective sleep quality is more commonly associated with sleep fragmentation (eg, WASO) than with TST.^43^

This study highlights the complex interplay between biopsychosocial factors and fatigue and sleep. Our findings align with previous longitudinal research that indicated an association between mood problems, higher levels of fatigue, and poorer subjective sleep quality.^14^,^44^ Participants with higher levels of general and physical fatigue consistently experienced more restrictions in participation over time. Managing symptoms that coexist with and influence fatigue and sleep quality, like anxiety and depression, is essential in rehabilitation, as these symptoms can greatly affect daily activity levels and overall health-related quality of life. Fatigue following TBI has been linked to slower information processing speed and impaired selective attention,^45^,^46^ which aligns with the trend we found showing an association between physical fatigue and slower processing speed, particularly in the acute stages post-injury. It is hypothesized that fatigue may result from impaired attention and speed of information processing, leading people with TBI to expend more effort on everyday tasks, thereby increasing fatigue.^8^

A strength of this study is the longitudinal design including 4 time points over the first 18 months post moderate-severe TBI, and the inclusion of different dimensions of fatigue and both subjective and objective measures of sleep. This study has several limitations. First, the small sample size limited our ability to identify distinct trajectories of sleep and fatigue, and prevented us from fully examining the intended biopsychosocial model that includes additional predictors such as pain, social support, and coping style. Additionally, multiple post-hoc comparisons were conducted within a small sample, highlighting the need for replication in a larger cohort. Second, there were missing data due to dropouts and participants starting the study at a later point. Furthermore, even for participants who completed all measures there was missing data on the SDMT due to the COVID pandemic. Third, the measures taken during the COVID-19 period may be less reliable due to the unique circumstances of the lockdown. Participants might have experienced fewer restrictions in participation since social activities, such as concerts or dinning out, were largely unavailable. Additionally, mood, fatigue, and participation levels could have been influenced by reduced social engagement during this period. Some participants may have felt better and less fatigued due to lower social pressures, while others may have experienced worsened outcomes due to increased isolation. Fourth, details about the injury severity, especially, LOC, PTA, GCS, were regularly missing in the medical files. As a result, we were unable to assess the contribution of these more robust biological factors to fatigue and sleep outcomes. Fifth, processing speed (SDMT), which was now used as the biological predictor, might have been affected by small learning effects. However, given that alternate forms were used and the assessments were spaced relatively far apart, the impact of learning effects is likely to have been minimal.^47^ Sixth, nearly 10% of participants had a history of concussion. While most individuals fully recover, we cannot rule out its potential influence on our results. Finally, this study may not generalize to the broader TBI population, as it included only individuals without premorbid conditions, whereas many people with TBI have comorbid psychiatric or neurological disorders that are risk factors for TBI.^48^,^49^ Furthermore, even though all participants qualified as moderate-severe according to the imaging results, it was a relatively mild sample according to the available PTA and LOC data.

In conclusion, this exploratory longitudinal study showed stable, and moderate to high levels of fatigue and poor sleep quality in the first 18 months post moderate to severe TBI. Physical fatigue and sleep duration decreased over time. Both fatigue and sleep were consistently associated with mood problems over time. Early mood interventions might aid the treatment for fatigue and sleep quality post-TBI. Moreover, the variability in symptom trajectories underscores the importance of individualized treatment approaches. Nonetheless, studies with larger sample are necessary to further explore the development and contribution of biopsychosocial factors to fatigue and sleep problems over time. A better understanding of the biopsychosocial factors associated with fatigue and sleep post-TBI at specific time points could improve the treatment of these symptoms.
